# The Jenner Society

**Published:** 1896-05-23

**Authors:** 


### EDITOR'S LETTER-BOX.

[Our correspondents are reminded that prolixity is a great bar to publication, and that brevity of style and conciseness of statement greatly facilitate early insertion.]

### THE JENNER SOCIETY.

We have received the following letter, enclosing several papers issued by the Jenner Society, from the hon. secretary, Dr. Francis T. Bond, of Gloucester:—With reference to the article of this week headed "Jenner: A Hundred Years After," I beg to enclose with this some evidence of the fact that, in spite of the melancholy illustration which the present epidemic of small-pox in Gloucester presents of the apparent forgetfulness by Jenner's native county of his beneficent work, those who really represent the intelligence of the county of Gloucester are determined that so far, at least, as they are concerned such a reflection shall be no longer applicable to them. How far the effort which they are making, the development of which has been temporarily suspended by purely accidental circumstances, will, when more fully realised, serve as a foundation for a wider recognition of Jenner's merits than are offered by his native county, must depend upon results which must be left to the future to demonstrate.

